# Urinary Podocalyxin-to-Creatinine Ratio as a Prognostic Biomarker of Renal Function Decline and Proteinuria Progression in Pediatric Chronic Kidney Disease

**DOI:** 10.3390/jcm15103762

**Published:** 2026-05-14

**Authors:** Nancy Lucero Martinez-Rodriguez, Miguel-Angel Villasis-Keever, Gabriela Alejandra Alegría-Torres, Jessie Nallely Zurita-Cruz, David Gregorio de Ita Pérez, Nadia Cruz-Ortega, Ramiro Alejandro Luna-Sánchez, Claudia del Carmen Zepeda Martínez, Alejandra Adilene Sánchez Chavelas

**Affiliations:** 1 Epidemiological Research Unit in Endocrinology and Nutrition, Hospital Infantil de México Federico Gómez, Ministry of Health (SSA), Mexico City 06720, Mexico; amr70@hotmail.com; 2 Analysis and Synthesis of the Evidence Research Unit, National Medical Center XXI Century, Instituto Mexicano del Seguro Social, Mexico City 06720, Mexico; miguel.villasis@gmail.com; 3 Department of Nephrology Pediatric, National Medical Center XXI Century, Instituto Mexicano del Seguro Social, Mexico City 06720, Mexico; gaby_ale1981@hotmail.com (G.A.A.-T.); deita_david@hotmail.com (D.G.d.I.P.); dra.nadia.cruz.ortega@gmail.com (N.C.-O.); drramiroluna@hotmail.com (R.A.L.-S.); cczmaat@hotmail.com (C.d.C.Z.M.); ales.chavelas@gmail.com (A.A.S.C.); 4 Research Division, Faculty of Medicine, Universidad Nacional Autónoma de Mexico (UNAM), Mexico City 04360, Mexico

**Keywords:** podocalyxin, urinary biomarkers, chronic kidney disease, pediatric nephrology, proteinuria, glomerular filtration rate, podocyte injury, disease progression

## Abstract

**Background**: Podocyte injury leads to the shedding of podocyte-derived molecules into the urine, which may serve as biomarkers of kidney disease. These molecules could be useful for the early detection of glomerular damage and for monitoring the progression of chronic kidney disease (CKD). **Methods**: A prospective cohort study was conducted in pediatric patients with CKD stages 1–4 treated at a tertiary care hospital between October 2019 and January 2023. Urinary podocalyxin and creatinine were measured at baseline. Renal function was assessed at baseline and at 12 and 24 months of follow-up. Patients were stratified by CKD stage. Changes in glomerular filtration rate (ΔGFR) were calculated, and correlations with baseline podocalyxin were evaluated using Spearman’s test. Multiple linear regression was used to adjust for confounders. **Results**: A total of 169 patients were included (median age 11 years). Glomerulopathies were the most frequent etiology (40.8%), and stage 1 was the most prevalent. At 12 months, stage 4 CKD patients showed a positive correlation with ΔGFR (r = 0.709, *p* = 0.003) and a negative correlation with proteinuria (r = −0.864, *p* < 0.001). At 24 months, a positive correlation was observed with ΔGFR (r = 0.949, *p* < 0.001), while an inverse association was observed with GFR. Associations varied according to CKD etiology. **Conclusions**: The podocalyxin-to-creatinine ratio was positively associated with renal function and negatively with proteinuria in stage 4 CKD, but showed no utility in stages 1–3.

## 1. Introduction

Chronic kidney disease (CKD) is a major non-communicable disease associated with substantial morbidity, mortality, and healthcare costs worldwide. It is defined as a persistent reduction in kidney function, characterized by a glomerular filtration rate (GFR) < 60 mL/min/1.73 m^2^ and/or markers of kidney damage lasting at least three months [1]. CKD affects more than 10% of the global population, and over 2.5 million individuals were receiving renal replacement therapy in 2015, a number projected to double by 2030.

In the pediatric population, CKD is a lifelong condition associated with substantial morbidity. Its etiology differs from that in adults, with congenital anomalies of the kidney and urinary tract and glomerular diseases being the most common causes [2]. Disease progression is driven by multiple factors, including proteinuria, hypertension, and podocyte injury, with proteinuria being a key marker and driver of disease progression [3,4,5]. Early diagnosis and timely intervention are critical; however, current clinical markers such as serum creatinine and albuminuria are limited in sensitivity and may not accurately reflect early renal damage [1].

The integrity of the glomerular filtration barrier depends on the coordinated function of endothelial cells, the glomerular basement membrane, and podocytes [6,7]. Podocytes play a central role in maintaining filtration selectivity, and their injury leads to structural disruption, protein leakage, and progressive loss of renal function [6]. Podocalyxin, a negatively charged transmembrane sialoprotein located on the apical surface of podocytes, contributes to maintaining cell architecture and charge selectivity within the filtration barrier [8]. Damage to podocytes results in the release of podocyte-derived molecules, including podocalyxin, into the urine, making them potential non-invasive biomarkers of glomerular injury [8,9].

Although proteinuria and estimated glomerular filtration rate remain the cornerstone markers for monitoring pediatric CKD, they do not fully capture ongoing structural kidney injury or explain the heterogeneity of disease progression among children [3,10]. Recent pediatric studies have therefore focused on urinary biomarkers of tubular injury, inflammation, repair, and glomerular damage, including NGAL, KIM-1, MCP-1, EGF, α1-microglobulin, nephrin, podocin, and podocalyxin [3,4,5,11,12,13,14]. However, evidence regarding urinary podocyte-specific biomarkers in pediatric CKD remains limited, particularly in longitudinal cohorts and across different CKD stages [3,10]. Podocalyxin was selected because it is highly expressed on the apical surface of podocytes, can be detected noninvasively in urine, and has been previously proposed as a marker of podocyte detachment or injury in glomerular diseases, including pediatric populations [3,4,5,11,12,13,14].

Several studies in adults have explored the diagnostic and prognostic value of urinary podocalyxin. Increased urinary podocalyxin levels have been associated with proteinuria, glomerular injury, and disease activity in conditions such as diabetic nephropathy and other glomerular diseases [12,15,16]. Additionally, the detection of podocyte-specific markers in urine has been linked to disease progression and renal function decline [17,18]. Despite these findings, most studies have focused on adult populations, and evidence in pediatric patients remains limited.

Given the need for reliable, non-invasive biomarkers capable of identifying disease progression at earlier stages, the evaluation of urinary podocalyxin and its normalization to creatinine may provide additional clinical value. However, studies assessing the podocalyxin-to-creatinine ratio as a marker of CKD progression in pediatric populations are scarce.

Therefore, the aim of this study was to determine the correlation between urinary podocalyxin and the podocalyxin-to-creatinine ratio with renal function decline and progression of proteinuria in pediatric patients with CKD stages 1–4.

## 2. Materials and Methods

### 2.1. Study Design

This prospective cohort study was conducted between January 2019 and December 2023 at a tertiary care pediatric center in Mexico (Pediatric Hospital, Centro Médico Nacional Siglo XXI). Participants younger than 18 years with a diagnosis of CKD stages 1–4, classified according to the National Kidney Foundation’s Kidney Disease Outcomes Quality Initiative (NKF-K/DOQI) criteria [19], were included. Exclusion criteria were the presence of macroscopic hematuria, refusal to participate, or loss to follow-up.

Data were collected on multiple variables, including anthropometric measurements, CKD etiology, and urinary podocalyxin levels at baseline. Renal function was assessed at baseline and at 12 and 24 months of follow-up. Renal function tests included creatinine clearance and 24 h urinary protein excretion.

### 2.2. Anthropometry

Height was measured with a stadiometer (SECA Model 769). Weight was measured with the bioimpedance method (Tanita BC-568 Segmental Body Composition Monitor, Tokyo, Japan) with the patient barefoot and wearing only underwear. Body mass index (BMI) was calculated by dividing the weight in kilograms by the height in meters squared, and then the percentile and BMI z-score were obtained according to age and sex. Classification of BMI was defined by the Centers for Disease Control and Prevention 2000, with children considered obese when their BMI for age and sex was in the ≥95th percentile, overweight when their BMI was >85th but <95th percentile, malnourished when their BMI was < 25th percentile, and normal weight when their BMI was within the 25th and 84th percentiles.

### 2.3. Serum and Urinary Biochemical Measurements

After a minimum of 8 h of fasting, blood samples were obtained from the antecubital vein. A 24 h urine collection was performed for the determination of creatinine clearance and proteinuria. Serum and urinary creatinine levels were measured using colorimetric enzymatic methods (Bayer Diagnostics, Puteaux, France). Podocalyxin levels were quantified using an enzyme-linked immunosorbent assay (ELISA) (Human Podocalyxin (PCX) ELISA Kit, Catalog No. MBS455296, MyBioSource, Minneapolis, MN, USA). Plates were read using an ELISA microplate reader (Labsystems Multiskan EX, MTX Lab Systems Inc., Vienna, VA, USA), and all measurements were performed in duplicate according to the manufacturer’s instructions. Intra- and inter-assay coefficients of variation < 7% were considered acceptable. A standard curve was generated for each assay.

### 2.4. Variables and Definitions

The study variables included renal function, proteinuria, and urinary biomarkers. Renal function was estimated using the glomerular filtration rate (GFR), calculated from creatinine clearance obtained through 24 h urine collection, using both urinary and serum creatinine levels, and adjusted for body surface area (m^2^), in accordance with the National Kidney Foundation’s Kidney Disease Outcomes Quality Initiative (NKF-K/DOQI) criteria. Patients were classified according to CKD stage based on these guidelines: stage 1 (GFR ≥ 90 mL/min/1.73 m^2^), stage 2 (60–89 mL/min/1.73 m^2^), stage 3 (30–59 mL/min/1.73 m^2^), and stage 4 (15–29 mL/min/1.73 m^2^). Proteinuria was quantified from 24 h urine collections and expressed as mg/m^2^/h.

Patients received standardized treatment during follow-up according to CKD stage and underlying etiology, based on current clinical practice guidelines, including KDIGO recommendations for CKD and glomerular diseases, as well as International Pediatric Nephrology Association guidelines. Management included renin–angiotensin–aldosterone system blockade (ACE inhibitors or angiotensin receptor blockers), blood pressure control, and treatment of CKD-related complications such as anemia, metabolic acidosis, mineral and bone disorders, and nutritional alterations. Given that treatment strategies are largely determined by CKD etiology and disease severity, adjustment for CKD etiology and stratification by CKD stage in the analyses partially account for treatment-related variability [20,21,22,23,24].

Patients were also categorized according to CKD etiology based on their underlying diagnosis, as documented in their clinical records, and classified into the following groups: glomerulopathies, tubulopathies, cystic or structural abnormalities, and uropathies. Proteinuria was quantified by measuring total protein in 24 h urine samples and expressed as mg/24 h.

Additionally, urinary podocalyxin levels and the urinary podocalyxin-to-creatinine ratio were determined at baseline. To assess disease progression, changes (delta, Δ) in GFR and proteinuria at 12 and 24 months were calculated as the difference between follow-up values and baseline measurements.

### 2.5. Statistical Analysis

The Kolmogorov–Smirnov test indicated a non-normal distribution of continuous variables; therefore, these variables are presented as median and interquartile range (IQR). Patients were stratified according to CKD stage.

Changes in glomerular filtration rate (ΔGFR) and proteinuria were calculated at 12 and 24 months of follow-up. GFR, proteinuria, and their respective changes were compared across CKD stages using the Kruskal–Wallis test.

Correlations between ΔGFR and Δproteinuria with baseline urinary podocalyxin levels and the podocalyxin-to-creatinine ratio were assessed using Spearman’s rank correlation test, stratified by CKD stage.

Multivariable linear regression models were constructed to assess the association between the urinary podocalyxin-to-creatinine ratio and changes in creatinine clearance and proteinuria at 24 months. Analyses were stratified by CKD stage and adjusted for age and CKD etiology. Given that treatment strategies in pediatric CKD are largely determined by the underlying etiology and disease severity, this approach partially accounts for treatment-related variability during follow-up.

A priori sample size estimation was performed based on an expected correlation coefficient of r = 0.57 between urinary podocyte markers and proteinuria, derived from previous studies [17]. Using Fisher’s Z transformation, and assuming a two-sided α of 0.05 and 80% statistical power, the minimum required sample size was calculated using the formula: N = ((Zα + Zβ)/C)^2^ + 3, where C = 0.5 × ln((1 + r)/(1 − r)).

Based on these parameters, the estimated minimum sample size was 22 participants per group, according to CKD stage stratification.

Statistical significance was defined as a two-sided *p*-value < 0.05. All analyses were conducted using STATA version 16 (StataCorp, College Station, TX, USA).

### 2.6. Ethical Considerations

The study protocol complied with the principles of the Declaration of Helsinki and was approved by the National Research and Health Ethics Committee of the Mexican Social Security Institute (IMSS) (registry number R-2020-3603-023). The parents/caregivers provided written informed consent, and each child provided assent.

## 3. Results

### 3.1. Patient Characteristics

A total of 193 patients with CKD were initially identified. Of these, 7 were excluded due to incomplete samples, 3 because they had stage 5 CKD, and 14 because they did not complete the 12-month follow-up required to assess creatinine clearance. A final sample of 169 patients with CKD stages 1–4 was included in the analysis (Table 1).

The median age was 11 years (IQR 7–13), with no sex predominance (female 46.7%, n = 79; male 53.3%, n = 90). The median BMI z-score was 0.34 (IQR −0.72 to 1.27). Most patients had an adequate nutritional status (68.1%, n = 115), followed by overweight/obesity (19.5%, n = 33) and undernutrition (12.4%, n = 21).

CKD stage 1 was the most frequent (58.6%, n = 99), followed by stage 2 (18.3%, n = 31), stage 3 (14.2%, n = 24), and stage 4 (8.9%, n = 15). The most common etiology was glomerulopathy (40.8%, n = 69), followed by cystic or structural abnormalities (25.4%, n = 43), uropathy (23.1%, n = 39), and tubulopathy (10.6%, n = 18). Within stage 4 CKD subgroup, most patients had glomerulopathy (80%, n = 12), while a smaller proportion had cystic or structural abnormalities (20%, n = 3).

Baseline GFR estimated from creatinine clearance was 102.0 mL/min/1.73 m^2^ (IQR 65.0–137.6), and median proteinuria was 3.3 mg/m^2^/h (IQR 2.1–6.7). Median urinary podocalyxin was 2.0 ng/mL (IQR 0.98–3.19), and the podocalyxin-to-creatinine ratio was 0.044 ng/mg (IQR 0.013–0.094). No statistically significant differences were observed across CKD stages for urinary podocalyxin (*p* = 0.901) or the podocalyxin-to-creatinine ratio (*p* = 0.458) (Table 2).

### 3.2. Twelve-Month Follow-Up

At 12 months, the median ΔGFR varied across CKD stages, with the greatest decline observed in stage 4 CKD (−11.3 mL/min/1.73 m^2^) (Table 2). In contrast, stage 1 patients showed a decline of −8.0 mL/min/1.73 m^2^, while stages 2 and 3 showed slight increases (1.0 [IQR −5.0, 9.9] and 2.5 [IQR −3.9, 9.6], respectively).

Regarding proteinuria, the greatest increase was observed in stage 4 CKD (4.8 mg/m^2^/h), whereas minimal changes were observed in the other stages (Table 2).

A positive correlation between the podocalyxin-to-creatinine ratio and GFR at 12 months was identified only in stage 4 CKD (r = 0.709, *p* = 0.003), whereas no significant correlations were observed in stages 1–3 (Table 3).

Similarly, a significant correlation was identified between the podocalyxin-to-creatinine ratio and ΔGFR at 12 months in stage 4 CKD (r = 0.961, *p* < 0.001), with no significant associations in other stages (Table 4).

No significant correlation was found between the podocalyxin-to-creatinine ratio and proteinuria at 12 months in stages 1–3. However, a significant negative correlation was observed in stage 4 CKD (r = −0.864, *p* < 0.001). Likewise, a negative correlation was identified between the podocalyxin-to-creatinine ratio and Δproteinuria in stage 4 CKD (r = −0.578, *p* = 0.023) (Table 4).

### 3.3. Twenty-Four-Month Follow-Up

At 24 months, the greatest decline in GFR was observed in stage 1 CKD (−11.0 mL/min/1.73 m^2^ [IQR −39.2, 0.70]), while stage 4 CKD showed a smaller decline (−6.2 mL/min/1.73 m^2^ [IQR −12.2, −0.23]) (Table 2).

Changes in proteinuria did not differ significantly across CKD stages (*p* = 0.257), although stage 4 showed the largest increase (25.0 mg/m^2^/h [IQR 0.0, 50.0]) (Table 2).

At 24 months, the podocalyxin-to-creatinine ratio was negatively correlated with GFR in stage 4 CKD (r = −0.606, *p* = 0.030), while no significant correlations were observed in the other stages (Table 3).

A strong positive correlation was observed between the podocalyxin-to-creatinine ratio and ΔGFR in stage 4 CKD (r = 0.949, *p* < 0.001), with no significant associations in stages 1–3 (Table 4).

No significant correlation was found between the podocalyxin-to-creatinine ratio and proteinuria at 24 months in stages 1–3. However, a strong negative correlation was observed in stage 4 CKD (r = −0.938, *p* < 0.001), as well as with Δproteinuria (r = −0.939, *p* < 0.001) (Table 4).

### 3.4. Analysis According to CKD Etiology

When analyzed according to CKD etiology, the podocalyxin-to-creatinine ratio showed heterogeneous associations. In patients with glomerulopathy (n = 69), a weak positive correlation with GFR at 1 year was observed (r = 0.329, *p* = 0.005), along with a positive correlation with ΔGFR (r = 0.433, *p* < 0.001).

In contrast, patients with cystic or structural abnormalities (n = 43) showed significant negative correlations with GFR at both 1 year (r = −0.612, *p* < 0.001) and 2 years (r = −0.662, *p* < 0.001), while correlations with ΔGFR and Δproteinuria were not significant.

Other etiologies, including tubulopathy (n = 18) and uropathy (n = 39), did not show consistent correlations with renal function decline, although strong associations with proteinuria were observed in some subgroups (Table 5).

A stratified multivariable linear regression analysis was performed to evaluate the association between the urinary podocalyxin-to-creatinine ratio and ΔGFR and Δproteinuria at 24 months. Models were stratified by CKD stage and adjusted for age and CKD etiology.

No significant associations were observed in CKD stages 1–3. In contrast, in stage 4 CKD, a higher podocalyxin-to-creatinine ratio was significantly associated with an increase in ΔGFR (β = 114.1; 95% CI: 112.0 to 116.2; *p* < 0.001) and a decrease in Δproteinuria (β = −487.3; 95% CI: −496.3 to −478.3; *p* < 0.001) (Table S1).

These findings suggest that the association between podocalyxin and renal outcomes is stage-dependent and becomes more evident in advanced CKD. However, given the limited sample size in the stage 4 subgroup, these results should be interpreted with caution.

## 4. Discussion

### 4.1. Principal Findings

The main finding of this study is that the urinary podocalyxin-to-creatinine ratio was associated with changes in renal function and proteinuria only in patients with advanced CKD (stage 4), whereas no significant associations were observed in earlier stages. These findings suggest that podocalyxin is not a sensitive biomarker for early detection of renal injury in pediatric CKD but may have potential utility in monitoring disease progression in advanced stages.

### 4.2. Biological Rationale and Comparison with Previous Studies

Podocalyxin is a key component of the podocyte glycocalyx and plays a fundamental role in maintaining the structural and electrostatic integrity of the glomerular filtration barrier. Podocyte injury leads to the release of cytoskeleton-derived components into the urine, which has been associated with proteinuria and decline in GFR [6,8,9,17].

In adult populations, several studies have consistently demonstrated that increased urinary podocalyxin levels are associated with CKD progression, particularly in secondary glomerulopathies such as diabetic nephropathy and lupus nephritis [1,12,15,16,25]. These findings support its role as an early biomarker of glomerular injury, often preceding conventional markers such as proteinuria and microalbuminuria [1,3,4,5,6,7,8,9,10,11,12,13,14,15,26].

However, our findings differ from those reported in adult cohorts. In our pediatric population, no correlation was observed between podocalyxin (or its ratio with creatinine) and renal function or proteinuria in early CKD stages (1–3). This suggests that, in early disease, podocyte injury may be insufficient to produce detectable levels of urinary biomarkers, likely reflecting limited structural damage at the glomerular level [2,27].

### 4.3. Potential Explanations for Pediatric–Adult Differences and Interpretation of Findings in Advanced CKD

Interestingly, in our study, a higher podocalyxin-to-creatinine ratio was associated with an improvement in GFR over time in patients with stage 4 CKD. This finding contrasts with previous studies in adult populations, where increased urinary podocalyxin levels have been consistently associated with disease progression and worse renal outcomes [16,17,18,27].

Several factors likely explain this discrepancy. First, pediatric CKD differs substantially from adult CKD in its underlying etiology, with a predominance of congenital and structural disorders rather than degenerative or metabolic conditions [27,28,29]. These differences could influence both the extent and the biological significance of podocyte injury. In addition, children likely exhibit a distinct disease trajectory, with greater potential for functional adaptation and response to therapeutic interventions compared with adults [27,28].

Second, all patients in our cohort were managed with standardized, guideline-based interventions aimed at slowing CKD progression, including renin–angiotensin–aldosterone system blockade and control of metabolic and hemodynamic factors. These therapies are known to reduce intraglomerular pressure and proteinuria, and may modify the relationship between podocyte injury markers and renal outcomes over time [17,27]. Thus, the observed associations may partly reflect treatment-related effects rather than a direct biological relationship.

Importantly, the stage 4 CKD subgroup was predominantly composed of patients with glomerular disease, which may have influenced the observed associations. Given that podocalyxin reflects podocyte injury, its behavior is more biologically plausible in glomerulopathies than in non-glomerular conditions such as structural abnormalities. This etiological distribution should be considered when interpreting the stage-specific findings.

Additional methodological considerations should also be taken into account. Regression to the mean could contribute to the apparent improvement in GFR, particularly in patients with advanced CKD and more extreme baseline values. Furthermore, measurement variability—especially related to 24 h urine collection and biomarker quantification—may have introduced additional dispersion in the observed associations.

Taken together, these findings suggest that the relationship between urinary podocalyxin and renal outcomes in advanced CKD is complex and likely influenced by multiple interacting factors. Rather than indicating a protective or causal relationship, these findings should be interpreted cautiously, the observed associations may reflect a dynamic and non-linear relationship between podocyte injury and renal function in advanced disease.

From a broader conceptual perspective, these findings should be interpreted within the evolving framework of biomarker integration in CKD. As highlighted by Provenzano et al., CKD is a multifactorial and systemic condition in which individual biomarkers rarely provide sufficient prognostic value when evaluated in isolation [29,30]. Instead, contemporary approaches emphasize the integration of biomarkers into multivariable risk models alongside traditional measures such as eGFR and proteinuria, as well as clinical and etiological factors. Within this context, the stage-dependent associations observed in our study could reflect the complex interplay between declining renal function, proteinuria, and systemic processes such as inflammation and endothelial dysfunction. This framework may also explain the variability of biomarker performance across CKD stages, as their clinical relevance is likely modified by disease severity and competing pathophysiological mechanisms [29,30,31]. Therefore, urinary podocalyxin should not be interpreted as an independent biomarker but rather as part of a broader, context-dependent biomarker network.

### 4.4. Clinical Implications

From a clinical perspective, our findings indicate that podocalyxin should not be considered a reliable early biomarker for CKD progression in pediatric patients. However, its association with changes in renal function and proteinuria in advanced stages suggests that it may serve as a complementary tool for monitoring disease evolution in patients with more severe CKD.

This distinction is particularly relevant in pediatric nephrology, where early identification of progression remains challenging and where biomarkers must be interpreted within the context of developmental and etiological differences.

### 4.5. Limitations and Future Directions

This study has several limitations that should be acknowledged. First, although the overall sample size was adequate, the number of patients decreased after stratification by CKD stage, particularly in stage 4 (n = 15), which may limit statistical power and increase susceptibility to variability in this subgroup. However, this distribution is consistent with the known epidemiological pattern of CKD, in which advanced stages represent a smaller proportion of the overall population, as described in the “iceberg” model of CKD prevalence [32]. This limitation is particularly relevant for the interpretation of subgroup analyses in stage 4 CKD and reinforces the need for cautious interpretation of these findings.

Second, urinary podocalyxin was measured only at baseline, whereas renal outcomes were assessed longitudinally over a 24-month follow-up period. This design limits the ability to establish temporal relationships or infer a true prognostic role, as biomarker levels may vary over time depending on disease activity and treatment. Therefore, the observed associations should not be interpreted as predictive.

Third, renal function was assessed using 24 h creatinine clearance, which, although routinely used in our institution and providing a direct measurement of kidney function, is subject to potential inaccuracies related to urine collection and may be less standardized compared with estimated glomerular filtration rate (eGFR) equations commonly used in pediatric populations. This may also limit direct comparison with studies that use standardized eGFR equations in pediatric populations.

Fourth, the observational design precludes establishing causal relationships between podocalyxin levels and renal outcomes, and although multivariable models were used to adjust for age and CKD etiology, residual confounding cannot be excluded and remains an inherent limitation of the observational study design. Additionally, although patients received standardized treatment, the potential influence of therapeutic interventions on longitudinal outcomes cannot be fully separated from the observed associations.

Fifth, the study included multiple subgroup and time-point analyses without formal correction for multiple comparisons, which may increase the risk of type I error. These findings should therefore be interpreted conservatively to avoid overinterpretation.

Additionally, the single-center design may introduce selection bias, and the relatively short follow-up period limits the ability to assess long-term clinical implications.

Further research is needed to validate these findings in larger, multicenter cohorts with repeated biomarker measurements over time to better define temporal dynamics and clinical utility. Future studies should also explore the role of podocalyxin in combination with other urinary biomarkers to improve risk stratification and early detection of glomerular injury.

Given the limited evidence in pediatric populations, expanding research in this area is essential, particularly considering that most available data are derived from adult populations with predominantly degenerative etiologies, which are not directly comparable to pediatric CKD.

## 5. Conclusions

Urinary podocalyxin does not appear to be a reliable early biomarker for detecting disease progression in pediatric patients with CKD stages 1–3.

In contrast, in advanced CKD (stage 4), the podocalyxin-to-creatinine ratio was associated with changes in renal function and proteinuria; however, these findings should be interpreted with caution given the limited sample size and the exploratory nature of the analysis.

Overall, our results suggest that the clinical relevance of urinary podocalyxin is stage-dependent and context-specific, and that it should not be interpreted as an independent biomarker but rather as part of a broader framework that includes clinical variables and traditional markers such as eGFR and proteinuria.

These findings highlight important differences between pediatric and adult CKD and underscore the need for age-specific approaches in biomarker research.

Future studies with larger cohorts, repeated biomarker measurements, and integration into multivariable models are required to clarify the clinical utility of urinary podocalyxin in pediatric CKD.

## Figures and Tables

**Table 1:** General characteristics of patients with chronic kidney disease (CKD) included in the study.

Variable	Statistics n = 169
Age (years), *median (IQR)*	11 (7, 13)
Sex, n (%)	
Female	79 (46.7)
Male	90(53.3)
BMI z-score, *median (IQR)*	0.34 (−0.72, 1.27)
Nutritional status, n (%)	
Undernutrition	21 (12.4)
Normal	115 (68.1)
Overweight/Obesity	33 (19.5)
CKD stage, n (%)	
1	99 (58.6)
2	31 (18.3)
3	24 (14.2)
4	15 (8.9)
CDK Etiology, n (%)	
Glomerulopathy	69 (40.8)
Tubulopathy	18 (10.6)
Cystic or structural abnormalities	43 (25.4)
Uropathy	39 (23.1)
GFR (mL/min/1.73 m^2^), *median (IQR)*	102.0 (65.0, 137.6)
24 h proteinuria (mg/m^2^/h), *median (IQR)*	3.3 (2.1, 6.7)
Urinary podocalyxin (ng/mL), *median (IQR)*	2.0 (0.98, 3.19)
Urinary podocalyxin-to-creatinine ratio (ng/mg), *median (IQR)*	0.044 (0.013, 0.094)

GFR: glomerular filtration rate.

**Table 2:** Podocalyxin and podocalyxin-to-creatinine ratio according to CKD stage based on GFR at baseline and follow-up.

		CKD Stage		
Variable	1 n = 99	2 n = 31	3 n = 24	4 n = 15
**Baseline (Median, IQR)**				
Proteinuria (mg/m^2^/h)	3.0 (2.2, 4.5)	3.8 (2.0, 4.5)	7.6 (1.4, 20.8)	25.0 (1.1, 25.0)
Podocalyxin (ng/mL)	1.9 (0.9, 3.3)	1.7 (0.3, 3.4)	1.9 (1.3, 2.5)	2.5 (1.2, 2.9)
Podocalyxin/creatinine (ng/mg)	0.04 (0.01, 0.08)	0.05 (0.01, 0.09)	0.05 (0.02, 0.10)	0.07 (0.01, 0.11)
**At 12-Month Follow-Up**				
ΔGFR (ml/min/1.73 m^2^)	−8.0 (−48.3, 10.3)	1.0 (−5.0, 9.9)	2.5 (−3.9, 9.6)	−11.3 (−13.0, 7.5)
ΔProteinuria (mg/m^2^/h)	−0.09 (−0.09, 0.60)	0.33 (−0.5, 1.02)	0.43 (−2.4, 2.3)	4.8 (3.4, 34.0)
**At 24-Month Follow-Up**				
ΔGFR (ml/min/1.73 m^2^)	−11.0 (−39.2, 0.70)	3.9 (−3.5, 12.9)	2.6 (−6.2, 18.5)	−6.2 (−12.2, −0.23)
ΔProteinuria (mg/m^2^/h)	0.06 (−1.8, 0.46)	0.93 (−0.93, 1.9)	0.66 (0.0, 4.2)	25.0 (0.0, 50.0)

GFR: glomerular filtration rate.

**Table 3:** Correlation between podocalyxin-to-creatinine ratio and GFR according to CKD stage at baseline, 12, and 24 months.

CKD Stage	Baseline		12 Months		24 Months	
	r	*p*	r	*p*	r	*p*
1 **(n = 99)**	−0.034	0.716	−0.005	0.960	−0.026	0.815
2 **(n = 31)**	0.085	0.631	0.028	0.877	−0.066	0.737
3 **(n = 24)**	−0.306	0.145	0.356	0.086	0.395	0.068
4 **(n = 15)**	−0.455	0.218	0.709	0.003	−0.606	0.030

GFR: glomerular filtration rate.

**Table 4:** Correlation between podocalyxin-to-creatinine ratio and ΔGFR and ΔProteinuria at 12 and 24 months.

Variable CKD Stage	12 Months		24 Months	
	r	*p*	r	*p*
**Podocalyxin/Creatinine Ratio**				
1 (n = 99)	0.083	0.411	0.019	0.861
2 (n = 31)	−0.188	0.309	−0.280	0.147
3 (n = 24)	0.229	0.281	0.286	0.195
4 (n = 15)	0.961	<0.001	0.949	<0.001
**Proteinuria**				
1 (n = 99)	0.091	0.367	0.086	0.338
2 (n = 31)	−0.114	0.538	−0.188	0.338
3 (n = 24)	0.007	0.974	−0.107	0.633
4 (n = 15)	−0.578	0.023	−0.939	<0.001

GFR: glomerular filtration rate.

**Table 5:** Correlation between podocalyxin-to-creatinine ratio and renal function according to CKD etiology.

Etiology	GFR (1 yr) r (*p*)	GFR (2 yr) r (*p*)	ΔGFR (1 yr) r (*p*)	ΔGFR (2 yr) r (*p*)	Proteinuria (1 yr) r (*p*)	Proteinuria (2 yr) r (*p*)	ΔProteinuria (1 yr) r (*p*)	ΔProteinuria (2 yr) r (*p*)
Glomerulopathy (n = 69)	0.329 (0.005)	−0.015 (0.906)	0.433 (<0.001)	−0.021 (0.871)	0.447 (<0.001)	0.073 (0.582)	0.056 (0.643)	−0.009 (0.941)
Tubulopathy (n = 18)	−0.073 (0.772)	0.205 (0.481)	−0.263 (0.291)	−0.104 (0.722)	0.927 (<0.001)	0.836 (0.002)	−0.120 (0.633)	0.359 (0.207)
Cystic/structural abnormalities (n = 43)	−0.612 (<0.001)	−0.662 (<0.001)	0.156 (0.317)	0.017 (0.916)	0.746 (<0.001)	0.701 (<0.001)	−0.012 (0.937)	0.001 (0.991)
Uropathy (n = 39)	−0.264 (0.103)	−0.195 (0.310)	0.185 (0.259)	−0.045 (0.812)	0.781 (<0.001)	0.948 (<0.001)	−0.150 (0.360)	−0.098 (0.610)

GFR: glomerular filtration rate.

## Data Availability

The data presented in this study are available upon request from the corresponding author due to privacy concerns.

## Supplementary Materials

The following supporting information can be downloaded at: https://www.mdpi.com/article/10.3390/jcm15103762/s1, Table S1. Linear Regression Analysis of the Association Between the ΔGFR and ΔProteinuria at 24 Months and Podocalyx-in-to-Creatinine Ratio.

## Abbreviations

CKD	Chronic kidney disease
GFR	Glomerular filtration rate
BMI	Body mass index
IQR	Interquartile Range
