# Supplementary material for: Urinary Podocalyxin-to-Creatinine Ratio as a Prognostic Biomarker of Renal Function Decline and Proteinuria Progression in Pediatric Chronic Kidney Disease
Source: J Clin Med. 2026 May 14;15(10):3762. doi: 10.3390/jcm15103762 (PMC13207470; doi:10.3390/jcm15103762)
Supplement: Supplementary file 1 [file jcm-15-03762-s001.zip › jcm-4292151-supplementary.pdf]

Supplementary Table S1. Linear Regression Analysis of the Association Between the ΔGFR and ΔProteinuria at 24 Months and Podocalyxin-to-Creatinine Ratio*

	Coef	95% CI		P
ΔGFR				
**Podocalyxin/Creatinine Ratio**				
CKD Stage				
1 (n=99)	1.3	-166.7	169.4	0.987
2 (n=31)	-97.8	-228.6	32.9	0.136
3 (n=24)	23.6	-14.6	62.0	0.212
4 (n=15)	114.1	112.0	116.2	<0.001
**ΔProteinuria**				
**Podocalyxin/Creatinine Ratio**				
CKD stage				
1 (n=99)	22.9	-41.1	87.1	0.478
2 (n=31)	-10.5	-39.0	17.9	0.454
3 (n=24)	-10.6	-54.2	33.0	0.616
4 (n=15)	-487.3	-496.3	-478.3	<0.001

*adjusted for CKD etiology and age

GFR: glomerular filtration rate
